# Effects of corn particle size on energy and nutrient digestibility in diets fed to young pigs and adult sows

**DOI:** 10.5713/ab.20.0556

**Published:** 2021-01-01

**Authors:** Dongli Ma, Tao Zhu, Fengjuan Yang, Shuai Zhang, Chengfei Huang

**Affiliations:** 1 State Key Laboratory of Animal Nutrition, College of Animal Science and Technology, China Agricultural University, Beijing 100193, China

**Keywords:** Adult Sows, Corn, Nutrient Digestibility, Particle Size, Young Pigs

## Abstract

**Objective:**

This research was carried out to investigate the effects of corn particle size on the apparent total tract digestibility (ATTD) of energy and nutrients fed to pigs at four different growth stages and therefore to provide basis for better application of corn in pig feeds.

**Methods:**

Eighteen weanling piglets, 18 growing barrows, 24 gestating sows and 24 lactating sows were used in this study. Within each stage, pigs were allotted to 1 of 3 or 4 corn-soybean meal diets which were formulated with different corn particle size in a completely randomized design with 6 replicate pigs per diet. Each stage lasted for 19 days, including 7 days for cages adaptation, 7 days for adaptation to diets and followed by 5 days for total collection of feces and urine.

**Results:**

For nursery and growing stages, the results showed that digestible energy content and ATTD of gross energy (GE), dry matter (DM), neutral detergent fiber (NDF), and acid detergent fiber (ADF) was increased (p<0.05) as the corn particle size reduced. Meanwhile, the metabolizable energy content and ATTD of crude protein (CP) tended to increase. For gestating sows, no differences were found in the ATTD of nutrients among dietary treatments. As for lactating sows, there were linear and quadratic increases (p<0.05) in the ATTD of DM, GE, NDF as the corn being finer milled. Quadratic response in ATTD of ADF and CP (p<0.05) were observed as sows fed with four different diets.

**Conclusion:**

Reducing corn particle size can increase digestibility of nutrients fed to young pigs and lactating sows. No effects were observed in present experiment when gestating sows were fed with different particle sized corn.

## INTRODUCTION

Feed cost accounts for a large proportion in the cost of livestock production and it determines the economic benefits of farming to a great extent. Therefore, how to reduce feed cost is an important challenge in animal production. Corn grain is widely used in animal feeds as carbohydrate or energy source due to its availability and good quality [1]. In recent years, the rising price of maize has also increased the cost of feeding. Thus, its necessary to improve the utilization efficiency of corn for the swine industry.

Processing procedure is one of the factors that will affect the nutritional value of corn. Corn grain will be milled to specify particle size before adding to the diets. Previous research had revealed that reduced particle size can improve nutrient digestibility [2]. At present, it is recommended grinding corn grain to a particle size of 640 to 650 μm [3]. However, Rojas and Stein [4] concluded that decreasing the particle size of corn from 865 to 339 μm linearly increased the concentration of digestible energy (DE), metabolizable energy (ME), and the apparent ileal digestibility (AID) of starch and gross energy (GE) in corn [4]. Thus, the optimal particle size of corn when fed to pigs is still unclear. Healy et al [5] observed that the optimal particle size of the feed ingredient increased with rising age of animal. This indicated that the optimal particle size of corn may be different when fed to pigs at different growing stages. But the available data for this part of the content is still relatively small.

Therefore, the objective of this research was to investigate the effects of corn particle size on energy and nutrient utilization efficiency of pigs at four different growth stages, and to provide basis for better application of corn in pig feeds.

## MATERIALS AND METHODS

All protocols used in this study were approved by the Institutional Animal Welfare and Use Committee of China Agricultural University (number AW10100202-2, Beijing, China). This study was conducted at Fengning Swine Research Unit of China Agricultural University (Academician Workstation in Chengdejiuyun Agricultural & Livestock Co., Ltd., Chengde, China).

### Ingredients sourcing and particle size processing

Corn used in this experiment was sourced from Beijing Baisilongda Technology Development Co., Ltd. (Beijing, China) and milled to specific particle size by a hammer mill (56×40, Muyang, Jiangsu, China) before inclusion into the experimental diets. Corn was crushed through 1, 2.5, 4, and 5 mm sieves to obtain corn with geometric average particle size of 400, 600, 800, and 1,000 μm. The test sieves (sieve size 2,360, 1,180, 600, 425, 300, 150, 106 μm and a solid metal pan) were stacked from the largest to the smallest mesh size. A sample of 100 g corn was placed on the top of the test screen and then this set were located in a Ro-Tap sieve shaker (Tyler Industrial Products, Mentor, OH, USA) for 10 min. After sieving, the residues on each sieve were weighed. The particle size was determined by the method described by American Society of Agricultural and Biological Engineers (ANSI/ASAE S319.4) [6]. The geometric mean diameter of the sample was calculated according to the aforementioned standard. Soybean meal used for all the experiment diets was ground in a 3.0-mm sieve.

### Animal, diets, and experimental design

Eighteen weanling piglets (Duroc×Landrace×Yorkshire, initial body weight [BW]: 10.2±0.4 kg), 18 growing barrows (Duroc× Landrace×Yorkshire, initial BW: 35.6±1.5 kg), 24 gestating sows (Landrace×Yorkshire, initial BW: 252±2.8 kg, parity 3; days 60 of gestation), and 24 lactating sows (Landrace× Yorkshire, initial BW: 241±3.8 kg, parity 3; days 10 of lactation) were allotted to 1 of 3 or 4 diets with 6 pigs per diet at each BW in a completely randomized design. During the experiment all pigs were fed with a corn-soybean meal diet and the only difference among diets was that the corn used in the diets was ground to specified particle size. Three particle sizes of corn (i.e., 400, 600, and 800 μm) for weanling and growing pigs and four particle sizes of corn (i.e., 400, 600, 800, and 1,000 μm) for adult sows. All the diets were formulated to meet or exceed the NRC (2012) recommended nutrient requirements [7]. Chromium oxide (2.5 g/kg) was added to gestating and lactating diets as an indigestible marker providing an alternative method for measuring energy and nutrient digestibility. All diets were given as mash and pigs had free access to water through a low-pressure nipple drinker. The ingredients and analyzed chemical compositions of experimental diets are shown in Table 1.

All pigs were housed in stainless-steel metabolism crates. Room temperature was controlled at 24°C to 27°C, 18°C to 22°C, 18°C to 22°C, and 22°C to 25°C, for weanling, growing, gestating and lactating pigs, respectively. Each stage lasted for 19 days, including 7 days for cages adaptation, 7 days for adaptation to diets and followed by 5 days for total collection of feces and urine. A standard corn-soybean meal diet was fed to pigs during the cage adaptation period. For weanling and growing pigs, the daily amount of feed was gradually increased until it was equivalent to 4% of their BW as described by Kong and Adeola [8], the feeding level of gestating and lactating sows adjusted to 2.5 and 5.0 kg/d, respectively. The daily feed allowance was divided into two equal meals and supplied at 08:30 and 15:30. Pigs were weighed at the beginning of the feed adaptation period and at the end of the feces and urine collection period. The amount of feed provided and leftovers were recorded at each feeding time to calculate daily feed consumption.

### Sample collection

All the feces and urine produced during the collection period were collected and stored in a −20°C refrigerator. Feces were collected twice daily at 08:30 and 15:30. Meanwhile, urine was collected into plastic buckets through a funnel installed under the metabolism cages. Approximately 50 mL of 6 *N* HCl was added to the buckets to limit microbial growth and to reduce the loss of ammonia. The volume of the urine excreted from each pig was recorded daily, and a sub-sample of 10% of the fully mixed urine was kept. At the end of the experiment, all kept feces and urine samples were thawed and thoroughly mixed. Approximately 350 g sub-sample of feces was taken and dried in a forced-draft oven at 65°C for 72 h. The feed and fecal samples were ground through a 60-mesh screen and then kept for further chemical analysis. Urine was filtered into 50 mL centrifuge tube used for follow-up determination.

### Chemical analysis

Feed and fecal samples were analyzed for dry matter (DM; method 934.01), crude protein (CP; method 990.03), crude ash (method 942.15), and phosphorus (method 985.01) [9]. Neutral detergent fiber (NDF) and acid detergent fiber (ADF) were determined following a modified procedure of van Soest et al [10]. Heat stable α-amylase and sodium sulfite were used for the analysis of NDF concentration without correction for insoluble ash as adapted for an Ankom Fiber Analyzer (Ankom Technology, Macedon, NY, USA). The concentration of ADF was analyzed in a separate sample. The GE of diets, fecal and urine were analyzed using an adiabatic oxygen bomb calorimeter (Parr Instruments Co., Moline, IL, USA). All chemical analyses were conducted in duplicate and repeated if the duplicates differed greater than 5%.

### Calculations

For weanling and growing pigs, the DE and ME contents of the diets were calculated according to the method followed the equations listed below:

$$\begin{array}{l} \text{The DE value of the diet }(\text{MJ}/\text{kg})=(\text{GE in feed intake},\text{MJ}-\text{GE in feces},\text{MJ})/\text{feed intake},\text{kg}\\ \text{The ME value of the diet }(\text{MJ}/\text{kg})=(\text{GE in feed intake},\text{MJ}-\text{GE in feces},\text{MJ}-\text{GE in urine},\text{MJ})/\text{feed intake},\text{kg.} \end{array}$$

The ATTD of nutrient was calculated using the equation: ATTDn (%) = [(Fin – Fout)/Fin] × 100%, where ATTDn is the apparent total tract digestibility of nutrient (%), Fin is the total intake of nutrient (g) from day 8 to 12, and Fout is the total fecal output of nutrient (g) originating from the feed that was fed from day 8 to 12.

For gestating and lactating stages, the ATTD of nutrient was calculated using the equation: ATTDn (%) = (1–NF/ND) ×(CD/CF)×100%, where NF and ND represent the nutrient concentration (%) in feces and diet, respectively and CD and CF represent the chromic oxide concentration (%) in diet and feces, respectively.

### Statistical analysis

All the data were checked for normality using UNIVARIATE procedure of SAS 9.1 (SAS, 2001) and then were analyzed with one-way analysis of variance using general linear model procedure of SAS. Polynomial contrast was also conducted using the CONTRAST statement to determine linear and quadratic effect of particle size using the interactive matrix language procedure of SAS. Pigs were treated as the experimental unit and the dietary treatment was the only fixed effect in the model. Statistical significance was declared as p<0.05.

## RESULTS

During the experiment, all pigs kept healthy and readily consumed their diets without any problems. The mean particle sizes of the milled corn were very close (+24 to −17 μm) to the aimed particle sizes (Table 2). The grinding process had achieved the expected effect.

The results for nursery pigs (Table 3) revealed that DE content and ATTD of DM, ADF (p<0.05), GE, NDF (linear and quadratic, p<0.05) were increased as the particle size reduced. Meanwhile, the ME content tend to be increased (p = 0.05). There was quadratic response in ATTD of CP (p<0.05) as pigs fed with different particle size of corn. For growing pigs (Table 4), the results showed that the DE content and ATTD of GE, DM, NDF, and ADF were linearly (p<0.05) increased as the corn particle size decreased from 800 μm to 400 μm. There was a tendency (p = 0.08) to increase ME content as corn particle size decreased. For gestating stage (Table 5), no improvements were observed in ATTD of energy and nutrients among pigs fed diets of four different treatments. For lactating sows (Table 6), the ATTD of DM, GE, NDF linear and quadratic (p<0.05) increased as the corn was finer milled. Quadratic response in ATTD of ADF and CP (p<0.05) were observed among dietary treatments.

## DISCUSSION

For young pigs, an increase of ATTD of energy and nutrients, along with DE and ME content were observed as the corn particle size reduced. This result is consisted with previous reports [4,11]. As reported before, reducing particle size by cereal grinding could increase the surface area for enzyme action and influence the gut fermentation and other digestive processes [12]. Moreover, fineness is associated with the increased production of hydrochloric acid and increased activity of pepsin in the stomach, which is essential for breaking down the protein matrix surrounding the starch granules [13]. It is well known that starch granules are tightly bound to proteins in the endosperm, which directly affects the digestion of total polysaccharides and the hydrolysis rate of digestive enzymes [14]. The opening of protein matrix is helpful to increase the contact between starch and α-amylase, which improves the digestibility of starch. In response, DE and ME content and ATTD of GE are increased. However, the decreased particle size did not improve the ME as much as the DE. This may be due to the finer corn treatment having a higher digestibility of CP and therefore, more protein was metabolized. Then, the urine nitrogen content and urine energy content were increased. As a result, ME was reduced more in these treatments than other treatments. Eventually, the improvement of ME was lower than DE. No differences were observed in ATTD of CP as the particle size decreased in this study. It seems that the contribution of CP from corn to the diet was not enough to change the digestibility of CP in the complete diet. Rojas and Stein [4] acquired similar results where AID and standardized ileal digestibility of CP and amino acids were not influenced by corn particle size [4]. This indicated that protein-digesting enzymes were not hindered by the reduced surface area and greater particle size in coarse ground corn. The improvements in ATTD of NDF and ADF might be associated with microbial fermentation in pigs. As reported before, the smaller particle size (430 μm or 450 μm or 470 μm) increased the total number of *Lactobacillus* sp. and *Bifidobacterium* sp. compared with the larger particle size (580 μm and 670 μm). These two kinds of bacteria are related to dietary fiber fermentation [15]. However, Fan et al [16] reported that the particle size of wheat diet did not affect the ATTD of GE, DM, organic matter, aNDF, and ADF. Zhao et al [17] expounded that the particle size had no effects on the ATTD of all chemical components of diets. These different results on ATTD of energy and nutrients may be attributed to the different chemical compositions of ingredients and different feed grinding degrees.

In present research, no improvements on the digestibility of nutrients for gestation sows were observed as the corn particle size decreased. According to the literature reviewed by the author, there are few experiments that studied the effect of corn particle size on nutrient digestibility of pregnant sows, therefore, there is basically no data to reference. Li et al [11] investigated the influence of brown rice particle size on nutrient digestibility for gestating sows. Inconsistent with the results herein, they found a reduction of particle size from 1,000 to 800 μm improved the ATTD of nutrient in diets. However, due to the different chemical compositions of the raw materials used in the research, these two results are not comparable. We have also noticed that the digestibility of nutrients was lower in gestating sows when compared with young pigs. It is reported that the digestibility of most dietary components improves as the pigs age and sows have a greater ability to digest nutrients compared with growing pigs [18]. One possible reason is the high level of crude fiber included in the experimental diets fed to pregnant sows. Previous literature revealed that increasing the dietary fiber content in diets will reduce the ATTD of nutrients and energy [19]. Meanwhile, the high level of dietary fiber decreased transit time of digesta in the gut, thus limited the time for microbial fermentation of digesta [20]. This can also reasonably explain why the digestibility of NDF and ADF is relatively low. Previous research supported this point as they reported that more insoluble and indigestible dietary fiber can reduce digestibility of NDF and ADF [21].

In current study, 400 μm exhibited the best performance when different comminuted corn was fed to lactation sows. This result agrees with Wondra et al [22], who reported a linear increase of apparent digestibility of nutrients as particle size of corn was decreased from 1,200 to 400 μm. Additionally, litter BW gain was increased by 11% as particle size was reduced from 1,200 to 400 μm in their research. It can be inferred that fine ground corn can affect the nutritional status of sows and then improve the growth performance of piglets. In present experiment, the digestibility of GE of lactating sows was improved by 5% as corn milled from 1,000 to 400 μm. Dietary energy concentration is important in maintaining BW of sows. Excessive weight loss during lactation will lead to prolonged interval from weaning to re-estrus [23,24]. From this point of view, the reduction of corn particle size is conducive to improving the efficiency of energy utilization, and thus improving the reproductive performance of sows. The reason for the increase in nutrients digestibility has already been discussed as above and will not be repeated here.

Comminution procedure is essential before corn mixed in feeds. It can maximize the utilization of corn. As shown in the results, the ATTD of nutrients was higher for young pigs with the particle size below 600 μm, and that 400 μm was the best for lactation sows. This conclusion is consistent with the previous reports. Lyu et al [25] recommended that milling corn to 618 μm is optimal in diets for 50 kg pigs. For weanling and growing pigs, pigs fed brown rice milled to 600 μm had greater ATTD of DM, GE, and CP than pigs fed brown rice ground to 800 μm and there were no further improvements in the ATTD of energy and nutrients as brown rice milled to 400 μm [11]. According to the results reported before, feed formulated with 400 μm corn has shown a higher digestibility of nutrients in lactation sows [22,26].

Regarding milling process, corn particle size and its distribution have technical and economic significance in pig production [14]. As previously reported, reducing particle size can increase surface area for enzyme action and thereby increase the digestibility of energy and nutrients [3,4]. Rojas et al [27] found pig dressing percentage linearly increased as the corn particle size decreased. They believed that a greater proportion of diet energy and nutrients is directed toward carcass tissue synthesis compared with synthesis of intestinal tissue. Paulk et al [28] reported a similar increase in dressing percentage as sorghum particle size was reduced. Although fine ground grain has many benefits to pigs, it does not mean that finer granularity is better. As the particle size decreases, many negative effects could emerge. Earlier research indicated that fine ground grains will induce ulcerations and keratinization when fed to pigs and ulceration is considered one of the major reasons for economic losses in the swine industry [22]. Healy et al [5] showed that with the reduction of grain size from 900 μm to 500 μm, feed production efficiency decreased and energy consumption increased. In addition, particles smaller than 400 μm may result in undesirable negative influences on gut health and inflammatory response [29]. However, due to the short experimental time, these parameters are not investigated in this study and further research is needed in the future.

## CONCLUSION

Reducing corn particle size could increase DE content and ATTD of DM, GE, NDF, and ADF, also tended to increase ME content and ATTD of CP for young pigs. For adult sows, the decrease of corn particle size improved the nutrients digestibility of lactating sows, but had no effect in gestating sows.

## Figures and Tables

**Table 1:** The ingredients and analyzed chemical composition of experimental diets (%, as-fed basis)

Item	Experimental diets			

	Nursery stage	Growing stage	Gestating stage	Lactating stage
Ingredients				
Corn	63.71	71.49	66.26	66.08
Soybean meal	23.30	25.62	13.30	24.61
Wheat bran	-	-	16.15	3.00
Fish meal	4.80	-	-	-
Soybean oil	0.60	-	-	1.07
Whey powder	5.00	-	-	-
Dicalcium phosphate	0.58	0.56	2.04	3.04
Limestone	0.70	1.03	0.73	0.52
Salt	0.25	0.30	0.25	0.25
L-Lysine-HCl, 98%	0.06	-	0.02	0.18
Chromic oxide	-	-	0.25	0.25
Premix^1)^	1.00	1.00	1.00	1.00
Chemical analyses				
Gross energy (MJ/kg)	16.06	16.14	15.10	15.75
Dry matter	88.95	88.65	86.27	89.20
Crude protein	19.58	18.26	14.22	17.81
Neutral detergent fiber	9.87	10.07	16.10	11.76
Acid detergent fiber	3.25	3.94	5.68	4.72
Calcium	0.70	0.60	0.75	0.89
Total phosphorus	0.57	0.51	0.60	0.62

1) Premix provided the following per kg of complete diet for nursery stage: vitamin A, 12,000 IU; vitamin D_3_, 2,500 IU; vitamin E, 30 IU; vitamin K_3_, 3 mg; vitamin B_12_, 0.012 mg; riboflavin, 4 mg; niacin, 40 mg; pantothenic acid, 15.0 mg; choline chloride, 400 mg; folacin, 0.7 mg; vitamin B_1_, 1.5 mg; vitamin B_6_, 3 mg; biotin, 0.1 mg; Zn, 105 mg; Mn, 22.0 mg; Fe, 84 mg; Cu, 225 mg; I, 0.50 mg; Se, 0.35 mg. For growing stage: vitamin A, 5,512 IU; vitamin D_3_, 2,200 IU; vitamin E, 30 IU; vitamin K_3_, 2.2 mg; vitamin B_12_, 27.6 μg; riboflavin, 4.0 mg; pantothenic acid, 14.0 mg; niacin, 30.0 mg; choline chloride, 400.0 mg; folacin, 0.7 mg; thiamine 1.5 mg; pyridoxine 3.0 mg; biotin, 44.0 μg; Mn, 40.0 mg; Fe, 75.0 mg; Zn, 75.0 mg; Cu, 100.0 mg; I, 0.3 mg; Se, 0.3 mg. For gestating stage: vitamin A, 5,512 IU; vitamin D_3_, 2,200 IU; vitamin E, 64 IU; vitamin K_3_, 2.2 mg; vitamin B_12_, 27.6 μg; riboflavin, 5.5 mg; pantothenic acid, 13.8 mg; niacin, 30.3 mg; choline chloride, 551 mg; Mn, 40 mg; Fe, 100 mg; Zn, 100 mg; Cu, 100 mg; I, 0.3 mg; Se, 0.3 mg. For lactating stage: vitamin A, 12,000 IU; vitamin D_3_, 2,000 IU; vitamin E, 24 IU; vitamin K_3_, 2.0 mg; vitamin B_1_, 2.0 mg; vitamin B_2_, 60 mg; vitamin B_6_, 4.0 mg; vitamin B_12_, 24 μg; niacin, 30 mg, pantothenic acid, 20 mg; folic acid, 3.6 mg; biotin, 0.40 mg; choline, 400 mg; Fe, 96 mg; Cu, 8.0 mg; Zn, 120 mg; Mn, 40 mg; I, 0.56 mg; Se, 0.40 mg, phytase, 600 FTU.

**Table 2:** Percentage of corn retained on sieves with different-sized openings and geometric mean (%, as-fed basis)

Item	Corn particle size (μm)			

	400	600	800	1,000
Sieve opening (μm)				
2,360	0.00	0.23	4.19	9.37
1,180	0.29	22.37	33.27	40.55
600	18.37	35.36	29.92	24.36
425	36.92	11.70	9.67	7.03
300	21.70	9.54	7.97	5.84
150	22.00	14.78	10.78	6.59
106	0.38	4.66	3.39	3.91
Residual	0.00	1.16	0.53	1.85
Geometric mean (μm)	424	620	783	985

**Table 3:** Effects of corn particle size on the apparent total tract digestibility of nutrients and energy values for nursery pigs

Item	Corn particle size (μm)			SEM	p-value		
	
	400	600	800		ANOVA	Linear	Quadratic
Digestibility coefficients (%)							
Gross energy	89.2	89.9	86.7	0.5	0.019	0.029	0.049
Dry matter	91.3	90.8	88.4	0.5	0.027	0.019	0.148
Crude protein	87.1	88.9	85.6	0.6	0.063	0.274	0.035
Neutral detergent fiber	66.4	73.5	54.8	2.5	0.002	0.017	0.003
Acid detergent fiber	64.5	73.3	64.0	1.7	0.032	0.885	0.010
Energy values (MJ/kg)							
Digestible energy	14.3	14.4	13.8	0.1	0.007	0.007	0.050
Metabolizable energy	14.1	14.1	13.6	0.1	0.050	0.027	0.266

Data are means of six observations.

SEM, standard error of the mean.

**Table 4:** Effects of corn particle size on the apparent total tract digestibility of nutrients and energy values for growing pigs

Item	Corn particle size (μm)			SEM	p-value		
	
	400	600	800		ANOVA	Linear	Quadratic
Digestibility coefficients (%)							
Gross energy	88.9	87.6	85.8	0.6	0.007	0.024	0.805
Dry matter	87.1	86.6	85.1	0.6	0.022	0.004	0.419
Crude protein	88.3	86.1	84.4	0.9	0.079	0.002	0.729
Neutral detergent fiber	66.6	62.3	54.4	1.0	0.002	0.016	0.324
Acid detergent fiber	64.6	63.8	62.0	1.3	0.031	0.012	0.425
Energy values (MJ/kg)							
Digestible energy	14.0	13.9	13.8	0.1	0.012	0.013	0.490
Metabolizable energy	13.7	13.4	13.3	0.1	0.081	0.162	0.577

Data are means of six observations.

SEM, standard error of the mean.

**Table 5:** Effects of corn particle size on the apparent total tract digestibility of energy and nutrients for gestating sows

Item	Corn particle size (μm)				SEM	p-value		
	
	400	600	800	1,000		ANOVA	Linear	Quadratic
Digestibility coefficients (%)								
Dry matter	78.7	79.2	78.2	76.6	0.5	0.192	0.699	0.524
Gross energy	79.8	79.7	78.8	78.6	0.5	0.274	0.505	0.787
Crude protein	81.2	82.1	80.4	77.1	0.7	0.353	0.716	0.470
Neutral detergent fiber	37.2	40.4	40.8	39.9	1.2	0.768	0.333	0.670
Acid detergent fiber	37.2	41.9	37.8	37.0	1.1	0.323	0.844	0.103

Data are means of six observations.

SEM, standard error of the mean.

**Table 6:** Effects of corn particle size on the apparent total tract digestibility of energy and nutrients for lactating sows

Item	Corn particle size (μm)				SEM	p-value		
	
	400	600	800	1,000		ANOVA	Linear	Quadratic
Digestibility coefficients (%)								
Dry matter	87.4	82.7	84.0	83.6	0.7	0.042	0.044	0.039
Gross energy	90.0	84.8	85.9	85.5	0.6	0.003	0.005	0.009
Crude protein	88.3	85.0	86.6	86.6	0.5	0.122	0.190	0.042
Neutral detergent fiber	75.5	57.4	61.9	58.2	2.5	0.017	0.024	0.028
Acid detergent fiber	72.6	57.4	62.6	61.4	2.1	0.047	0.062	0.031

Data are means of six observations.

SEM, standard error of the mean.
